# The influence of manual semen collection in male trained dogs (*Canis familiaris*), in the presence or absence of a female in estrus, on the concentrations of cortisol, oxytocin, prolactin and testosterone

**DOI:** 10.1371/journal.pone.0278524

**Published:** 2023-02-02

**Authors:** Martyna Woszczyło, Antoni Szumny, Piotr Knap, Tadeusz Jezierski, Wojciech Niżański, Agata Kokocińska, Marcin J. Skwark, Michał Dzięcioł

**Affiliations:** 1 Department of Reproduction, Wroclaw University of Environmental and Life Sciences, Wrocław, Poland; 2 Department of Chemistry, Wroclaw University of Environmental and Life Sciences, Wrocław, Poland; 3 Department of Epizootiology and Clinic of Birth and Exotic Animals, Wroclaw University of Environmental and Life Sciences, Wrocław, Poland; 4 Institute of Genetics and Animal Biotechnology, Polish Academy of Sciences, Department of Animal Behavior and Welfare, Magdalenka, Poland; 5 Institute of Biological Bases of Animal Production, University of Life Sciences in Lublin, Lublin, Poland; 6 InstaDeep Ltd., London, United Kingdom; Sathyabama Institute of Science and Technology, INDIA

## Abstract

Sex pheromones are chemical substances secreted into the environment that affect the physiology and behavior of recipients. Females use these compounds during oestrus to attract males, which leads to attempts of mating. This study evaluates the influence of manual semen collection in male dogs, in the presence or absence of a female in estrus, on the blood concentrations of cortisol (CRT), oxytocin (OXT), prolactin (PRL) and testosterone (T), as hormones involved both in the physiology of reproduction and stress. Ten male dogs were used in Experiment 1 to measure the serum and plasma concentrations of the aforementioned hormones in the absence of semen collection. Subsequently in the same animals, the concentrations of these hormones were evaluated before and after semen collection in the presence (Exp. 2) or in absence of a female in estrus (Exp. 3). No significant changes in hormone concentration caused by the semen collection were found, either with, or without the presence of female in estrus. Obtained results suggest that the procedure of manual semen collection in dogs, probably due to its passive character, does not stimulate endocrine glands to secrete hormones, and the process of ejaculation is probably controlled by neural pathway. The lack of effect of semiochemical stimulation to the CRT, PRL, OXT and T level, could be caused by a short contact with female during semen collection. Further studies on involvement of the hormones during the process of natural mating, especially preceded by long courtships, similar to that observed under natural conditions, should shed a light on the physiology of mating and the connection between the endocrine system and semiochemical stimulation in dogs.

## Introduction

Semen collection from male dogs is routinely performed in clinical practice, e.g. for artificial insemination, cryopreservation, evaluation of semen quality and fertility assessment. In contrast to humans, where the hormonal profile associated with sexual activities such as masturbation, orgasm, coitus, and ejaculation has been well described, there is little to no high-profile research on the impact of manual semen collection on the hormonal profile in trained dogs. [1].

Animal physiology changes in the presence of stressor to allow organism to adjust to challenging environmental conditions and maintain homeostasis. Observed reactions include changes in behavior, autonomic nervous system, neuroendocrine system, and immune system [2]. Stress response in animals occurs mostly in two pathways–fast reaction of autonomic nervous system (sympatho-adrenomedullary system–SAM), with signs of stress including changes in heart rate or blood pressure, as well as prolonged reaction of neuroendocrine system, when stressor activates hypothalamic–pituitary–adrenal (HPA) axis, which in turn leads to the release of glucocorticoids [2–5]. In addition, the activation of the neuroendocrine cascade is caused by the experience of agitation and arousal, which is associated with the receipt of positive stimuli [3]. The level of stress factors as well as the ability of the animal to challenge it, seem to be factors enabling discrimination between the distress and eustress. It is due to the stressful factors possibly leading to adaptation, and thus consequently improving animals’ welfare or even enabling their survival [3].

In the past, cortisol (CRT), which is produced by the adrenal gland, was thought to be the primary reference marker for stress in relation to the activation of the HPA axis. However, a recent study revealed that prolactin (PRL) also plays a role in the stress response in dogs [6]. This could suggest that the stress response in dogs is associated with increased levels of both hormones. In addition to the role of PRL in the stimulation and maintenance of lactation, this hormone plays an important role in many other physiological processes and in some species, its release was found to accompany ejaculation [1, 6–8]. In humans, PRL seems to be responsible for the feedback control of sexual drive and rises after orgasm (both in masturbation and coitus conditions) [1, 6, 8]. This hormone is also known as "parental hormone"[9], as it plays crucial role in both maternal and paternal behavior in both human and other mammals [10]. In biparental mammals, prolactin may not be a "hormone of paternity" even though it is elevated in fathers of several mammals [11]. However, prolactin promotes paternal behavior in uniparental rats (*Rattus norvegicus*) [12].

While there have been reports of a meaningful role played by other glucocorticoids, such as corticosterone, in the mating behavior of other species (e.g. rats) [13], their role in dogs has yet to be fully explored. Prior work, if considering corticosterone, tends to aggregate its levels with these of cortisol [14]. In the interest of clarity we have omitted the problem of corticoid metabolism in this work, but we do acknowledge that both cortisol and corticosterone are products of closely related synthetic pathways (with cortisol being a hydroxylated corticosterone) and could have comparable importance in dogs. It is not the case with humans, but it is in rats, birds and reptiles. We posit that it is unlikely that corticosterone is of a high importance in dog mating behavior, but this remains to be ascertained.

Oxytocin (OXT), a neuropeptide produced in the hypothalamic paraventricular and supraoptic nuclei, in female plays a role in the milk secretion and uterine contraction, as well as being involved in the development of maternal behavior (mother-neonate bond) [15]. It is involved in various behavioral reactions, including orgasm, social recognition, pair bonding or anxiety [16–19]. Intranasal administration of OXT may influence serum and urine OXT levels [20–22] and potentially influences some dogs behavior [20, 22–24]. Moreover, it was shown that plasma OXT levels in dogs increase during social interactions with humans [25–27]. This hormone is also widely considered to be a putative physiological indicator of welfare [28].

In the reproductive physiology of male rodents, OXT plays a role in erection, copulatory activity and ejaculation [29]. Injection of oxytocin receptor antagonist attenuates ejaculation and affects pre-ejaculatory behavior during normal sexual activity. There is evidence that a group of oxytocin neurons located in the posterior paraventricular nucleus of the hypothalamus (PVN) projects to the lower spinal cord and controls penile erection and ejaculation in male rats [30].

In humans, during erection and sexual arousal OXT, together with dopamine and norephinephrine, plays paramount role as a neurotransmitter. An increased secretion of OXT, as a consequence of orgasm, has been previously described [31, 32]. Concentration of this hormone increases after ejaculation in men to by 20 to 360%, and decreases to the baseline concentration after approximately 10 minutes [33].

In some animals, such as stallions and boars, an increased blood concentration of OXT after semen collection was also reported [34]. Additionally, the content of OXT in the saliva of boars was shown to be higher during the collection of their semen [35]. Even though OXT levels have been measured in connection with the process of collecting semen from dogs, this analysis has mostly concentrated on the quality of the semen, and has not looked at male behavior or stress [16, 36]. In the context of testosterone (T) after semen collection, its rise has been observed in stallions [37]. However, for dogs, previous study demonstrates discordant results. For instance, Fucuda et al. [38] noticed increased serum T concentration after male dogs had contact with secretions from females in estrus, but not after direct contact with female in estrus. In a stressful situation, the blood supply to the testicles decreases (for example in male rats [39]), and as a result of chronic stress the level of testosterone in blood can be lowered. Literature describes also a phenomenon of a temporary, initial increase in the level of this hormone under the influence of stress, dependent on the social status of the individual: individuals with a higher social status were less sensitive to lowering testosterone levels under the stressful circumstances [40].

Animals react differently to stressful situations, if they are repeated. Upon repeated exposures to a potentially stressful situation, a process of either habituation or sensitization may occur. Sensitization leads to an increased response to a stimulus, and is more likely to occur in the case of aversive stimuli that are of high intensity or low predictability. On the other hand, habituation is a form of non-associative learning that results in decreased responsiveness to repeated stimuli [4, 5, 41–43]. During habituation, both behavioral reaction decreases and hypothalamic–pituitary–adrenal (HPA) activation decrease, which occurs in response to a stressor. In addition, animals can modify their response to the stressful event, in the mechanism of counterconditioning and desensitization [41]. Desensitization depends on habituation, so the dog gradually becomes accustomed to an increasing stimulus. In counterconditioning, a stressful stimulus is linked to food or other reinforcement. This makes the initially stressful stimulus a harbinger of something pleasant for the animal, reducing its distress (but not necessarily its arousal) [43].

Pheromones are the chemical compounds secreted by the emitter to the environment that modify behavior as well as physiology of the signal recipient. Those substances are used by females in estrus to attract male which results finally in attempts of mating [44]. Semen collection in dogs, on the other hand, is frequently done without the natural semiochemical and visual stimulation provided by the presence of a female in estrus. The effect of this procedure on the male’s physiology and welfare is not yet fully understood. The aim of the present study was to address this gap in knowledge, by investigating the influence of presence of a female in estrus and, possibly, sex pheromones themselves on the endocrine status of a male, and evaluation the influence of semen collection in the presence or absence of a female in estrus, on the circulating levels of CRT, OXT, PRL and T as hormones involved in the processes of reproductive physiology and indicating welfare status of animals.

One cannot discount the role of cognitive factors in the arousal of the animal, where sole presence of the potential partner may elicit a response, due to prior mating experiences. This aspect is difficult to control for, even in the tight regime of experimental conditions, as presented in this work. We do posit, however, that these experiences can be treated as latent variables, and do not significantly affect the validity of the conclusions.

## Materials and methods

### Ethical statement

The research was carried out in accordance with the regulations on animal experimentation and the guidelines for the use of animals in research. The 1^st^ Local Committee for Experiments with the Use of Laboratory Animals, Wroclaw, Poland issued a statement approving the experimental protocol used in this study (permission no. 105/2017, 088/2021/P1). Due to the fact that all research was conducted on the grounds of the University of Life Sciences in Wroclaw, involving Beagle dogs from a local research kennel, no further licenses were necessary beyond the previously mentioned approval for the use of animals in the experiment.

### Animals

In the experiment, 10 adult intact male Beagle dogs from a local research kennel (aged 1–7 years, body weight 10–14 kg) and two adult Beagle females (aged 2–3 years, body weight 10–12 kg) were used. All male dogs have experienced natural mating and manual semen collection. The dogs were kept in an outdoor kennel, in separate pens, 2–3 dogs together. Females were kept separately in other parts of the building. Male dogs were accustomed to the females used in experiment and had contact with them during anestrus.

All the dogs were accustomed to blood collection, they were previously subjected to habituation and counterconditioning training. Dogs were familiar to the person collecting the blood. The experiments took place in a well-ventilated and air-conditioned room, free from distracting odors, under similar temperature and humidity conditions. The feeding, playing and walks schedule was appropriately adjusted in order not to interfere with the experiments.

### Experimental setup

**Fig 1:**
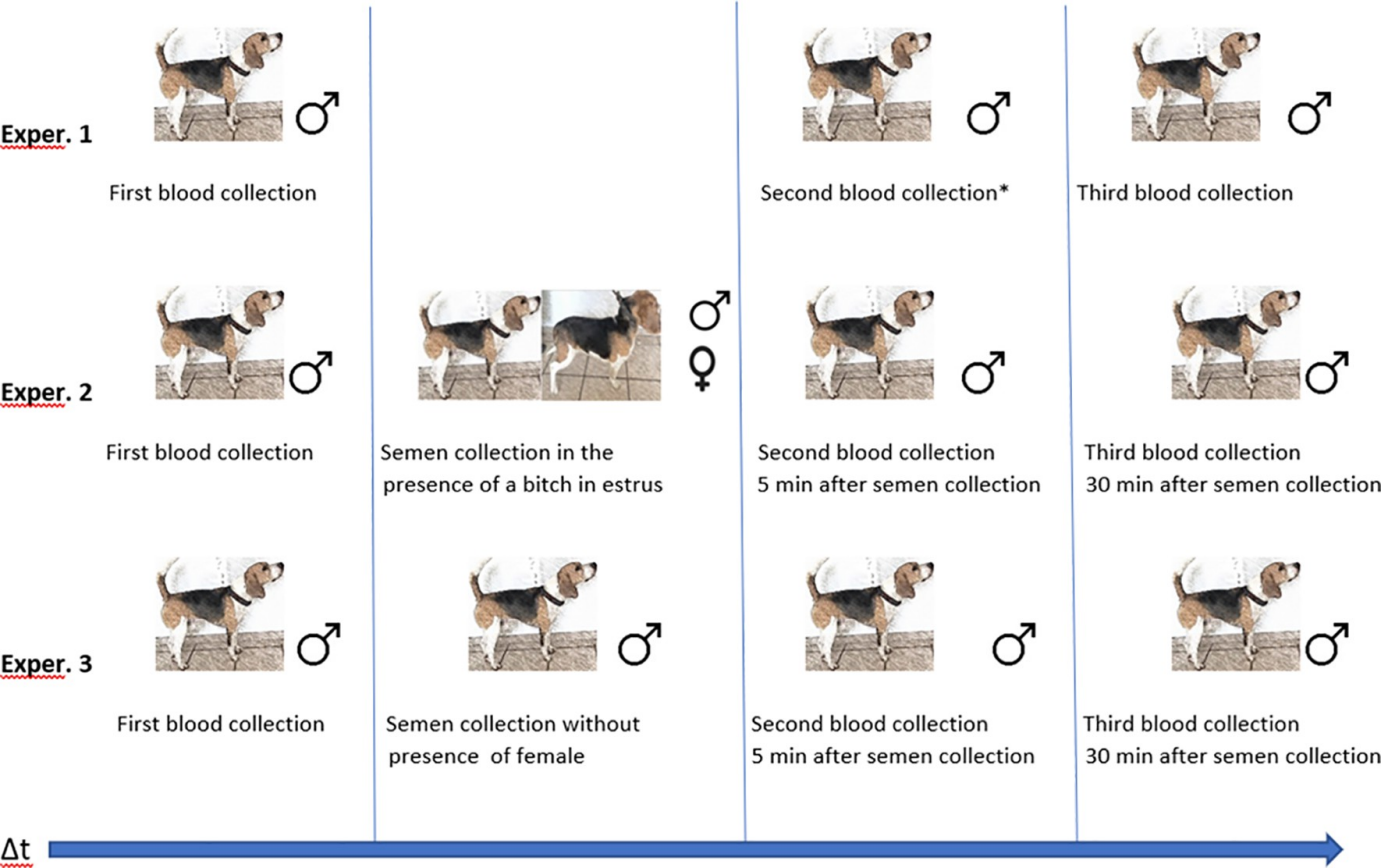
Experiment design-scheme of blood (exp. 1, 2, 3) and semen collection (exp. 2, 3), with (exp. 2) or without (exp. 3) presence of the female in estrus. *in all experiments blood was collected in the same time intervals.

Three main experiments were conducted according to the scheme presented in Fig 1. In the first experiment (Exp. 1), the basal concentrations of studied hormones as well as their dynamics during the time planned for the experiment were evaluated. The main aim of this experiment was to evaluate their baseline levels, since there is a natural variation in concentration of these hormones throughout the day [45–48]. In addition, since the same time intervals between blood collections were to be used in the subsequent experiments, it was necessary to determine whether the procedure of collecting blood may have any effect on the assessed hormone levels. Blood was collected one hour before semen collection (baseline concentration) and then five minutes and thirty minutes after semen collection, therefore intervals between samples were as follows: one hour and fifteen minutes between sample 1 and 2, and thirty minutes between sample 2 and 3.

During Experiment 1, the dogs were not exposed to any procedure connected neither with semen collection, nor with female visual or pheromonal stimulation. Interactions between handlers and dogs were limited to a minimum.

In Experiment 2, blood samples were collected at three time points: one hour before semen collection (basal concentration), and 5 minutes and 30 minutes after semen collection. Semen collection was performed in the presence of a female in estrus, confirmed by the evaluation of vaginal cytology, estimation of the serum level of progesterone and full behavioral signs of estrus [49, 50]. Male dogs were allowed close, direct contact with the female, including sniffing and licking the vulva, however, no mating was allowed. Dogs were allowed to maintain direct contact with the female for the whole time when semen was collected, which took approximately five minutes.

In Experiment 3, semen and blood were collected exactly at the same time intervals as in Experiment 1 and 2, but in the absence of a female in estrus. During the blood and semen collection, the male dogs were standing on the floor and all manipulations were performed by the same handler, with whom the dogs were familiar with.

Experiments were conducted always at the same time during the day. In Experiment 1, 2 and 3 first blood samples were collected at the same time in the morning, second and third blood samples were collected at the same time intervals to avoid fluctuations of hormone levels with time. All 10 dogs were used in each of the three experiments. The studies were conducted early in the morning. Dogs had fasted for at least eight hours. The cycle of light and dark was normal.

No additional experimental group involving semen collection in the presence of a female in anestrus was used. This was explained by the fact that in contrast to other species (e.g. cattle) in dogs, females in anestrus are not usually used for male stimulation. Our clinical experience as well as observations from a preliminary study showed that the presence of a female in anestrus during semen collection is not useful and only increased stress in both male and female. Due to the lack of the estrus specific pheromone signal and probably due to some specific semiochemical signals secreted by female in anestrus which was suggested in our previous studies [51], the presence of a female in anestrus could rather be thwarting than simulating the male’s sexual arousal during semen collection. In addition, the stress in a female can cause severe avoiding reaction, including even aggressive behavior both from the female and male, when not presenting an acceptance reflex.

#### Semen collection procedure

Semen was collected by the manual method [16]. Initially, each dog’s penis was massaged through the prepuce until a partial erection developed. Then, the prepuce was retracted past the bulbus glandis and firm pressure was applied to the penis behind the bulbus glandis. In all parts of the study the semen collecting procedure was performed without any disturbances and all three fractions of sperm was collected. Procedure took less than five minutes in each cases. In all cases semen volume was approximately 3 ml. Visual examination of the semen showed no abnormalities. More sophisticated methods of evaluating semen quality and quantity was not implemented, since this was not the purpose of the experiment. Dogs were standing on the floor and wasn’t restrained during the semen collection in other way than the leash and the collar.

#### Blood collection

Blood samples were collected by venipuncture of the cephalic vein, into anticoagulantfree tubes for serum (Separmed ®, F.L. Medical S.r.l., Torreglia, Italy), and into prechilled EDTA + Aprotinin tubes (BD Vacutainer®, BD, USA) for plasma to assess OXT concentration. The blood samples were centrifuged at 4°C and 1,200 Xg to recover plasma. The following step involved freezing 600 ul of serum and plasma from each sample and storing them at a temperature of -20°C until the hormone assay was completed [52]. During the blood collection procedure dogs were standing on the ground, venipuncture took less than a minute, and the whole procedure in the presence of the dog—including setup and transportation took less than five minutes.

Dogs in the study had their blood collected always by the same person, under the same conditions and in a reasonably short period of time. They were used to this procedure on the basis of counterconditioning training, but no reinforcements were used during the course of the experiment.

### Assessment of hormone concentration

#### Cortisol assay

CRT concentration was analyzed using the Vidas Cortisol S assay, Ref. 30 451, BioMérieux (Marcy-l’Étoile, France) [53].

#### Oxytocin assay

Plasma OXT was assessed using the EIA kit initially developed by Assay Designs Inc. and currently provided by Enzolifesciences, (Farmingdale, New York), ADI-901-153A-0001. Sensitivity range of the test is 15 pg/ml (dynamic 15.6–1,000 pg/ml). This assay is commonly used to evaluate OXT concentration in animal plasma and has been previously validated for use with dogs plasma [52]. OXT was extracted according to the manufacturer’s instructions, using the solid phase extraction (SPE) protocol with the following modifications: SPE C18 columns were 500 mg (Bionacom SPE C18 Cartridges), thus a larger volume of acetonitrile (2ml) was used to equilibrate them. Samples were eluted slowly (gravity-fed) by applying 3 mL of a solution comprised of 60% acetonitrile prepared using 0.1% TFA (Trifluroacetic acid) (49). Eluates were collected into 15 ml Falcon tubes for drying. Samples were dried in a vacuum centrifuge concentrator at 4°C for 6 hours. OXT concentration was measured immediately after reconstitution. The method to measure oxytocin concentration in plasma was executed in accordance with manufacturer instructions. All applied modifications were mentioned in the manuscript.

#### Prolactin assay

PRL was assessed using the enzyme immunoassay for the quantitative measurement of canine PRL in serum: Prolactin canine ELISA, nr DEV9944, Manufacturer/Supplier: Demeditec Diagnostics GmbH Lise-Meitner Straße 2 D-24145 Kiel. The test was performed according to the manufacturer’s instructions, without modifications [6]. Standard range of the assay is 2.5–80 ng/ml and sensitivity 0.4 ng/ml. This test is highly specific for canine prolactin. Detectable cross reactivities to other hormones that may be present in serum samples are not known.

#### Testosterone assay

Testosterone concentration was analyzed using the ELFA; mini VIDAS® Testosterone Assay, Ref: 414320, Biomerieux, (Marcy-l’Étoile, France).

### Statistical analysis

In order to address the research questions, we carried out statistical analysis using IBM SPSS Statistics 25 software. We calculated basic descriptive statistics (w.r.t. assay results at different time points and measurement regimes, as shown in Table 1) with Shapiro-Wilk test and two way mixed model ANOVA, comparing the significance of the difference in measured hormone levels across different measurement regimes. The alpha value threshold of .05 (*p* < .05) was considered as statistically significant.

**Table 1:** Basic descriptive statistics.

		*M*	*Me*	*SD*	*Sk*.	*Kurt*.	*Min*.	*Max*.	*K-S*	*p*
				Control						
Cortisol (ng/ml)	base	18.09	14.70	13.39	1.35	1.83	4.90	48.22	.87	101
	5 min	18.17	13.70	12.00	1.46	2.16	7.11	45.71	.84	.049
	30 min	20.96	17.47	12.31	1.02	1.07	6.13	47.34	.92	.338
Oxytocin (pg/ml)	base	24.60	21.30	9.86	0.97	0.26	13.00	44.50	.91	.288
	5 min	23.99	25.00	9.16	-0.09	-1.51	11.20	35.30	.90	.247
	30 min	32.26	28.40	13.03	1.15	1.07	17.50	60.30	.89	.183
Prolactine (ng/ml)	base	4.38	2.11	4.25	0.58	-1.62	0.00	11.20	.84	.049
	5 min	5.12	3.72	4.46	0.27	-2.04	0.23	11.43	.84	.047
	30 min	4.40	3.57	3.55	0.54	-0.85	0.00	10.74	.94	.572
Testosterone (ng/ml)	base	1.22	0.57	1.34	1.59	1.29	0.23	4.01	.73	.001
	5 min	2.80	2.48	1.83	0.32	-1.31	0.54	5.61	.93	.435
	30 min	2.09	1.96	1.68	0.19	1.94	0.21	4.65	.84	.049
Semen collecton with female										
Cortisol (ng/ml)	base	8.33	7.18	6.22	2.06	5.23	2.49	24.18	.78	.009.
	5 min	7.98	7.78	5.44	1.66	4.34	1.00	21.35	.84	039
	30 min	12.28	12.14	4.25	0.31	-0.45	6.30	19.64	.97	.870
Oxytocin (pg/ml)	base	29.55	30.03	13.68	1.21	3.16	12.30	61.40	.85	.066
	5 min	30.33	29.45	12.63	0.73	0.00	13.30	53.40	.93	.468
	30 min	32.50	29.15	11.72	1.81	4.18	19.90	61.40	.83	.035
Prolactine (ng/ml)	base	4.28	2.32	4.22	0.97	-0.52	0.00	12.12	.86	.068
	5 min	4.79	2.82	4.39	1.25	0.34	0.57	13.77	.80	.012
	30 min	4.62	2.12	4.17	0.99	-0.49	0.98	12.55	.82	.023
Testosterone (ng/ml)	base	1.18	0.72	1.14	1.15	-0.06	0.14	3.20	.81	.019
	5 min	2.21	2.04	1.44	0.23	-1.32	0.21	4.38	.95	.615
	30 min	1.48	0.66	1.77	1.85	2.84	0.29	5.61	.72	.001
Semen collecton without female										
Cortisol (ng/ml)	base	9.71	8.22	2.95	0.61	-1.45	6.56	14.11	.85	.055
	5 min	12.27	11.38	5.89	1.19	1.09	6.62	24.92	.88	.129
	30 min	10.62	9.04	4.39	0.93	0.14	5.82	19.54	.89	.173
Oxytocin (pg/ml)	base	22.81	21.40	7.39	0.87	0.62	12.20	37.20	.92	.365
	5 min	27.07	24.78	13.85	2.46	7.07	13.30	64.10	.70	.000
	30 min	23.63	24.90	7.42	-0.15	-1.08	13.00	34.80	.95	.689
Prolactine (ng/ml)	base	4.36	1.77	5.31	1.88	3.54	0.00	17.25	.76	.004
	5 min	4.94	2.45	4.54	1.04	-0.88	1.14	12.64	.75	.003
	30 min	4.96	2.69	4.97	0.88	-1.02	0.16	13.41	.82	.025
Testosterone (ng/ml)	base	2.30	1.43	2.82	2.07	4.38	0.16	9.32	. .74	. 002
	5 min	2.13	0.89	3.22	2.77	8.07	0.09	. 10.96	60	< .001
	30 min	2.51	2.25	1.99	0.88	0.41	0.17	6.43	.92	.355

M—mean

Me—median

SD—standard deviation

Sk.—Skewness

Kurt—kurtosis

Min—minimum

Max—maximum

K-S—result of Kolmogorov—Smirnov test

p—p-value

In context of the subsequent sections, in line with common nomenclature in multivariate research [54], we consider “main effect” as the effect of an independent variable on a dependent variable averaged across the levels of any other independent variables. We consider *interaction* (and corresponding *interaction effect*) as a statistical coupling between two independent variables (effect of one independent variable on another). Finally we consider “simple effect” to be the effect of an *independent variable within one level of another independent variable*.

#### Descriptive statistics

Descriptive statistics were shown in Table 1. Shapiro-Wilk test showed that distribution of results in several variables can for all intents and purpose be considered normal. Small sample size precludes conducting more involved statistical analysis, hence estimated confidence intervals cannot be considered to be entirely dependable. However, skewness in all samples were smaller than the absolute value of 3, so parametrical test could be performed [55].

## Results

### Cortisol level

Statistically significant group main effect was found, *F*(1; 27) = 5.42; *p* = .010; η^2^ = .29, which clearly indicates higheffect size. Therefore, Sidak *post-hoc* tests were performed. Significant differences were found between control group (*Mean* = 19.07; *SE* = 2.22) and semen collection with female group (*Mean* = 9.53; *SE* = 2.22); *p* = .016, and between control group and semen collection without female group (*Mean* = 10.87; *SE* = 2.22), *p* = .043. The difference between two experimental groups was not statistically significant, *p* > .1.

Main effect of samples time collection was not statistically significant, F(2; 54) = 1.75; p = .184; η2 = .06. Also, no statistically significant interaction effect was found, F(4; 54) = 0.80; p = .532; η2 = .06. Nonetheless, simple effect analysis was performed.

**Fig 2:**
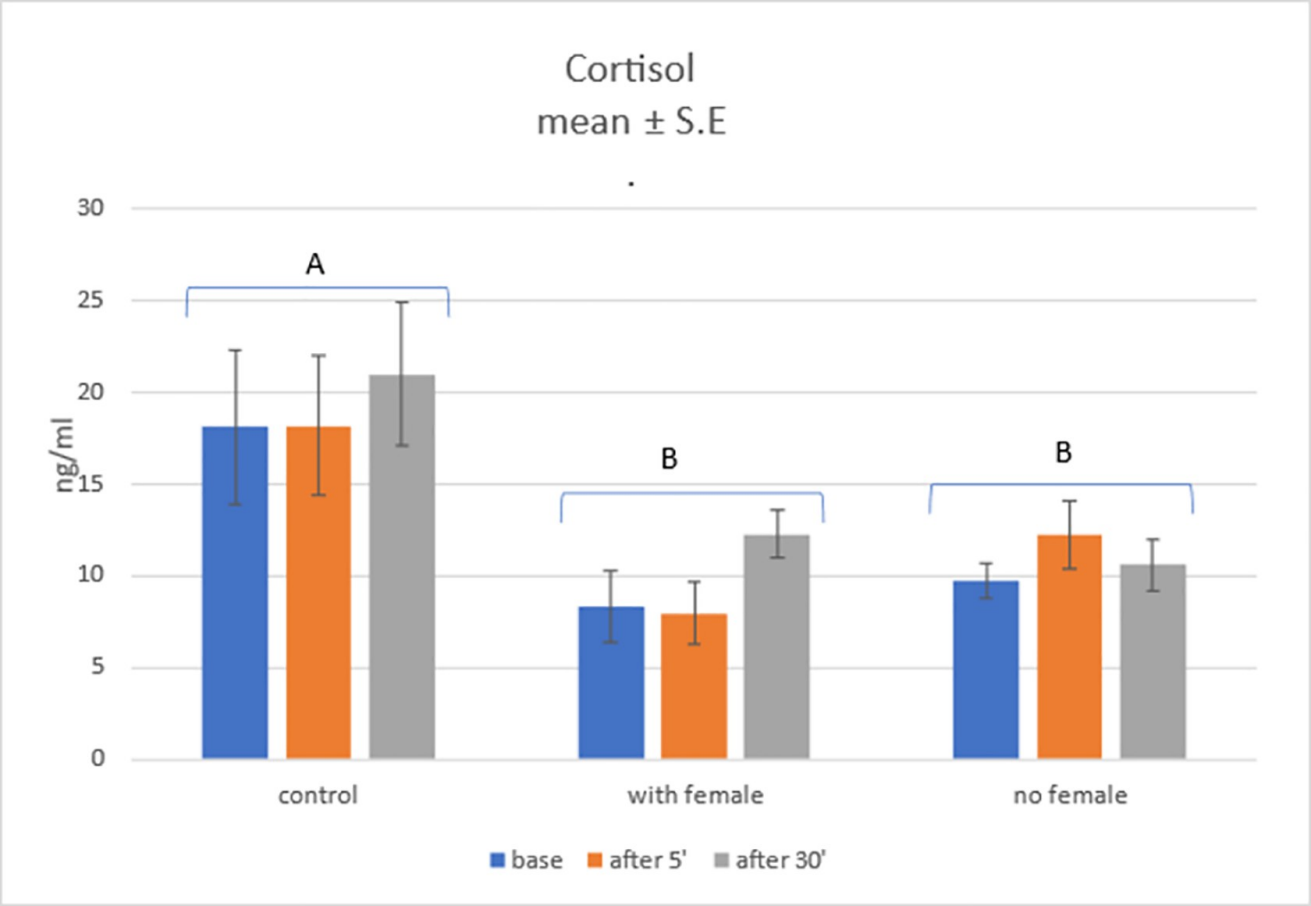
Cortisol level in three groups in three time points. A and B shows the statistical significant difference between the control and groups where semen was collected with and without presence of female in estrus.

We found statistically significant simple effect of group in all three time points: base, *F*(2, 27) = 3.69; *p* = .038; η^2^ = .22; 5 minutes, *F*(2, 27) = 3.77; *p* = .036; η^2^ = .22 and 30 minutes, *F*(2, 27) = 4.89; *p* = .015; η^2^ = .27. Sidak *post-hoc* tests were performed. In basal time collections point there was statistical trend between control group and semen collection with female group (*p* = .054). Sidak *post-hoc* test indicated no significant difference between control group and semen collection groups with or without female. In blood collections 5 minutes after semen collection statistically significant difference between control group and semen collection with female group was found (*p* = .032). Semen collection group without female was not statistically significantly different from two other groups (*p* > .1). In blood collections point at 30 minutes after semen collection there was significant difference between control group and semen collection without female group (*p* = .021). There was also nearly significant difference between control and semen collection with female groups (*p* = .063). Difference between two experimental groups was no statistically significant (*p* > 1). Results are shown in Fig 2. These results, at a threshold of significance, strongly point toward the need of conducting confirmatory assays, with a larger cohort of experimental subjects, to fully ascertain the role of presence of a female during the semen collection. Such an assay is outside the scope of this work and as such is to be a subject of future research.

### Oxytocin level

Statistically significant group main effect was found, *F*(2; 27) = 3.92; *p* = .032; η^2^ = .23. Size effect was high. Therefore, Sidak *post-hoc* tests were performed. Significant difference was found between semen collection with female group (*Mean* = 30.79; *SE* = 1.60) and semen collection without female group (*Mean* = 24.5; *SE* = 1.60); *p* = .029, Differences between control group (*Mean* = 26.95; *SE* = 1.60) and two experimental groups were not statistically significant, *p* > .1.

**Fig 3:**
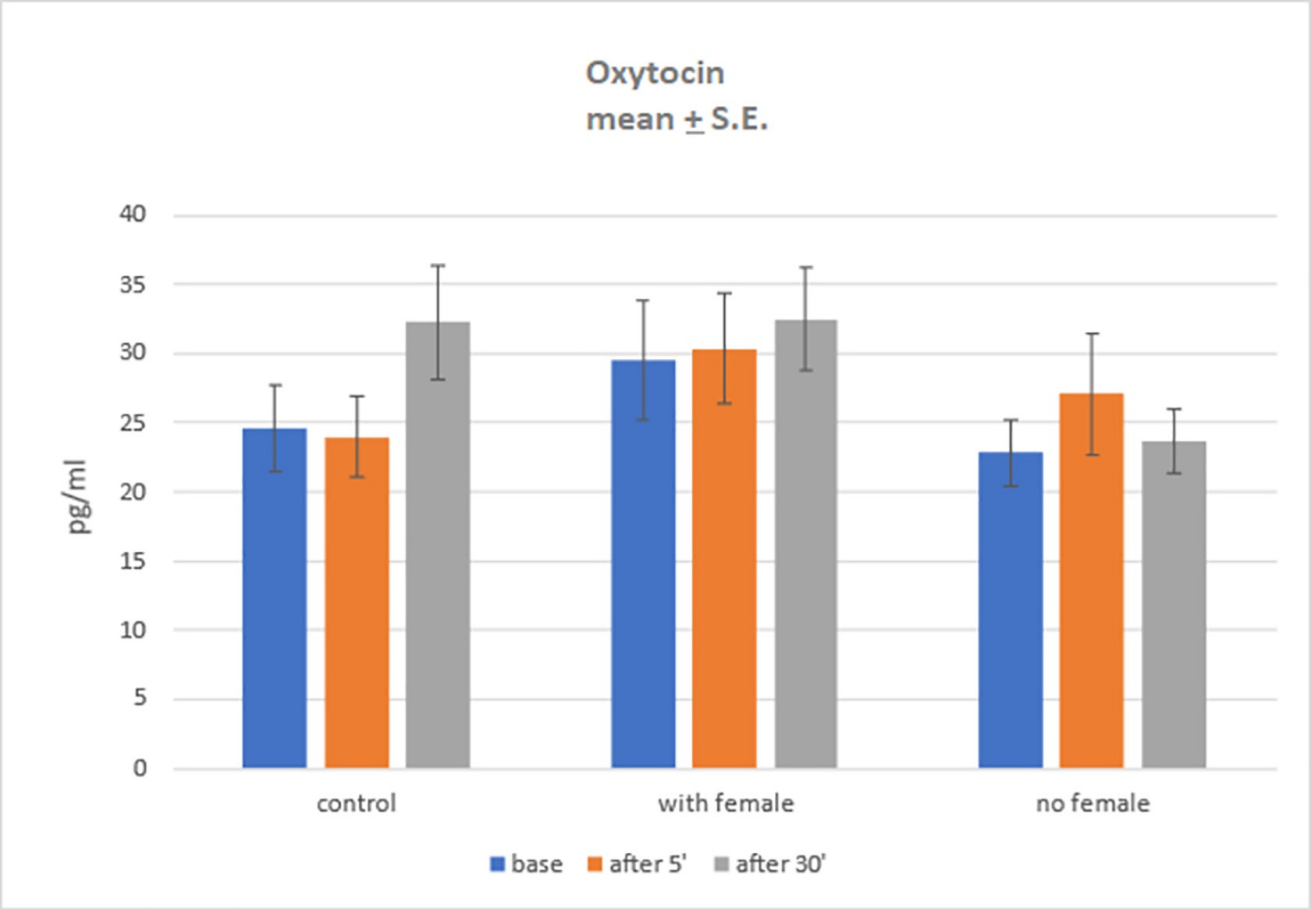
Oxytocin level in three groups in three time points.

Main effect of samples time collection was not statistically significant, *F*(2; 54) = 0.73; *p* = .485; η^2^ = .03. Also, no statistically significant interaction effect was found, *F*(4; 54) = 0.58; *p* = .675; η^2^ = .04. Nonetheless, simple effect analysis was performed but no statistically significant results were found. Results are shown in Fig 3. Due to the lack of significant, measurable difference between the control group and the experimental groups in terms of oxytocin levels, we cannot conclusively ascertain the role of female presence on the oxytocin level during semen collection.

#### Prolactin level

**Fig 4:**
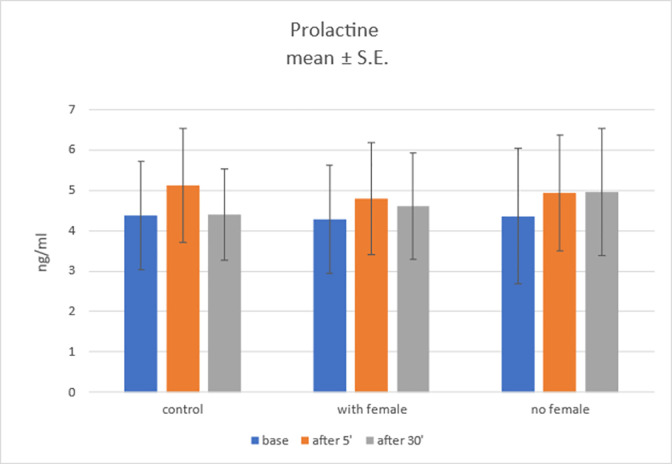
Prolactin level in three groups in three time points.

Group main effect was found to not be statistically significant, *F*(2; 27) = 0.01; *p* = .995; η^2^ = 0. Main effect of samples time collection was not statistically significant either, *F*(2; 54) = 1.35; *p* = .268; η^2^ = .05. Also, no statistically significant interaction effect was found, *F*(4; 54) = 0.20; *p* = .937; η^2^ = .02. Nonetheless, simple effect analysis was performed but, no statistically significant results were found here either. Results are shown on Fig 4. We conclude that in light of our experiment there are no grounds to ascribe prolactin level fluctuations to the mode of semen collection.

#### Testosterone level

Group main effect was found not statistically significant, *F*(2; 27) = 0.42; *p* = .659; η^2^ = 0.30 Main effect of samples time collection was marginally statistically significant, *F*(2; 54) = 2.72; *p* = .075; η^2^ = .09. These results did not allow for use of *post-hoc* tests to determine the differences between base (*Mean* = 1.57; *SE* = 0.35), 5 minutes (*Mean* = 2.38; *SE* = 0.42) and 30 minutes time point (*Mean* = 2.03; *SE* = 0.33). Also, no statistically significant interaction effect was found, *F*(4; 54) = 1.21; *p* = .318; η^2^ = .08. Nonetheless, simple effect analysis was performed.

**Fig 5:**
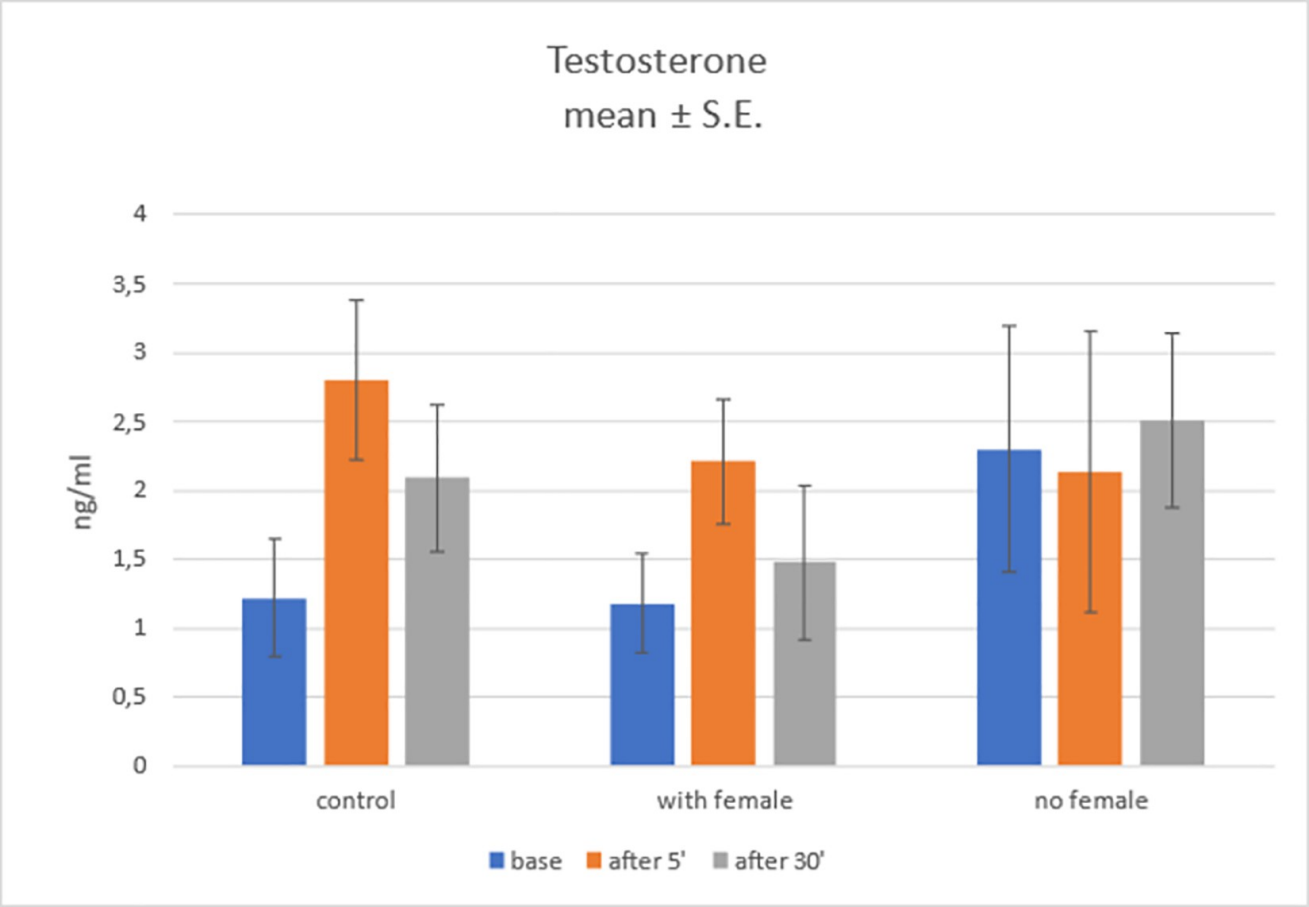
Testosterone level in three groups in three time points.

We found statistically significant simple effect of samples time collection in control group, *F*(2, 27) = 3.93; *p* = .032; η^2^ = .23. Therefore, Sidak *post-hoc* tests were performed. One statistically significant difference was found–between base and 5’ time point (*p =* .026). The differences between 30 minutes time point and two other samples time collection points were not statistically significant (*p* > .1). No other significant simple effects were found. Results are shown on Fig 5. Also in this case the statistical evidence for the influence of the mode of sample collection is not sufficient to draw conclusive inferences.

## Discussion

Blood sampling by venipuncture can be stressful and can increase the concentration of CRT shortly after the procedure. In such a case, a significant increase in CRT above the basal level can persist for up to 30 minutes [26, 56]. However, it was found that in dogs that are accustomed to venipuncture, there may be no increase in serum CRT concentration [57].

In animals, semen collection has been evaluated in the context of animal welfare, mostly in relation to the electroejaculation procedure [58–60]. Electroejaculation as a painful procedure, is very likely to be a source of stress and thus impacts the welfare. Moreover, changes in endocrinological status as a consequence of semen collection using artificial vagina have been evaluated in males of several species (e.g. horses, cattle, donkeys and pigs [35, 61–64]).

In most animal species, including dogs, cortisol (CRT) is considered the major indicator of an altered physiological state in response to stress. Although the concept of stress is usually associated with negative emotions (distress), sometimes a similar physiological response can be elicited by positive emotions associated with a desired and pleasant activity or situation (eustress). Archetypically, sexual arousal can be regarded as a source of eustress. In such a situation, the release of glucocorticoids and catecholamines can induce positive feedback and allow for increased activity and higher efficacy of the reflexes. This can explain the rise of CRT observed in studies, where semen was collected from stallions with the use of methods that mimicked natural mating behavior [34]. In humans however, where CRT was also measured after semen collection, the results seem to be discordant: Purvis et al. [65]reported an increased serum CRT concentration, whereas Krüger et al. [7]did not confirm this finding.

Semen collection using artificial vagina enables expressing full natural reproductive behavior repertoire [34, 37, 61, 63, 66]. On the contrary, in dogs, semen is collected by intensive mechanical stimulation, often without natural stimuli (either semiochemical or visual stimuli from a female in estrus). Thus, the artificial character of this procedure could interfere with the normal sexual behavior of a dog and may have impact on its welfare. In the context of our research, the mechanical procedure of semen collection could be a potential source of stress. On the other hand, contact with presenting acceptance reflex and secreting luring semiochemicals female leads to physiological sexual arousal, and potentially could be a source of eustress, and sex pheromones, implicated in such a scenario, could be considered to be natural reinforcements [51, 67].

This hypothesis could be further supported by the fact that in the analysis comparing the level of cortisol 30 minutes after semen collection in the group that did not have contact with the female in estrus, the level of cortisol was considerably lower than in the control group (p = .021). However the same cannot be stated regarding the test that was performed during the collection of semen in the presence of the female (p = .063). In light of the fact that cortisol levels fluctuate on a daily basis, this conclusion, which is based on the data that was presented above, should be considered with caution, as it needs further exploration.

The findings indicate that the manual collection of semen does not sufficiently stimulate the endocrine system to induce an increase in the level of hormones that are secreted above what is considered normal. Furthermore, a short-term exposure to female sexual pheromones has little impact on the levels of hormones found in the blood.

Comparing complete groups to one another may not provide accurate results due to the fluctuating amounts of cortisol that dogs experience on a daily basis, as well as variations in the concentration of this hormone that occur between various days. The most relevant observation would be the difference between the samples taken on a particular day when analyzing the influence of a given factor on cortisol levels. Nevertheless, in case of the experimental groups assessed in this work, we were unable to identify statistically significant variations between the samples.

Weak stimulation of HPA axis and CRT release in Experiments 2 and 3 suggests that manual semen collection in the dog can be considered a passive procedure, as the penis is manually stimulated by the handler. Thus, the lack of significant sexual stimulation from a female and of the need for active sexual behavior could be considered as a reason for the lack of an adequate response from the adrenal gland, expressed as a surge in CRT during semen collection. Therefore, in the case presented in this study, a lack of significant increase in CRT and PRL may indicate that sufficient excitement is not elicited. However, no significant changes in CRT and PRL concentrations may also be indicative of the effects of habituation [68].

Regardless of the fact that we observed a group effect, as there was a significant difference in oxytocin level between semen collection with and without a female in estrus, the level of oxytocin in plasma after semen collection did not differ from that found in the control group in our study. Observed results were not consistent with these of Carmichael et al. [69], but in accordance with the findings of Huynh et al. [70], who concluded that orgasm in women activates the pituitary gland, while ejaculation in males does not cause this kind of effect. This observation confirms the previously mentioned low-key stimulation of the male dogs during manual semen collection. The inability to express a full range of mating behavior, resulting from the unnatural character of manual semen collection, could also be considered insufficient for the activation of the pituitary gland. Thus, we conjecture that in ejaculation in dogs during manual semen collection mainly the neural pathway is involved.

Traas and Kustritz [36] hypothesized that the presence of a bitch in estrus during semen collection may influence semen quality by increasing the sperm concentration. However, the findings of mentioned research do not support this claim. Neither the administration of OXT nor the semiochemical stimulation had any discernible effect on the quality of the sperm. It was stated by the authors that "it is not known if smooth muscle in the male canine reproductive tract contains receptors for (…) oxytocin (..)", despite the fact that "it has been shown that receptors for these hormones exist in the reproductive tract of the male mouse and boar" [29]. Considering correlation between OXT and CRT it is worth mentioning that Uvnäs Moberg et al. [71] stated that long-term release of OXT may have stress-reducing effect, as a consequence of a changed function of mineralocorticoid receptors (MR) and glucocorticoid receptors (GR) in the hippocampus, and decreased production of corticotropin releasing factor (CRF). However, Burri et al. [72] did not confirm that CRT concentration would decrease due to increased concentration of OXT.

PRL has been found to be indicative of both acute and chronic stress. Since all three hormones considered in our study (OXT, CRT and PRL) are related to stress reaction in dogs, they can be perceived as welfare markers. However as far as canine welfare is concerned, it must be taken into account that the context of sexual arousal (eustress) may interfere with the effect of potentially stressful procedure of collecting semen by manual masturbation. Assessment of welfare during particular activity or procedure, on the basis of hormones that are involved in regulation of the animal’s response to this activity at a normal physiological level that are related to distress e.g. involvement of oxytocin or prolactin in ejaculation, must be considered carefully because of the risk of misinterpretation. In our study, no significant influence of the procedure of manual semen collection on the profile of PRL was noted. Thus none of hormones evaluated in our study and involved in stress (eustress /distress) responses (CRT, OXT, PRL) did change significantly during manual semen collection. This could be explained by the fact that dogs used in our experiment were familiarized both with blood sampling and semen collection and indicates non-stressful behavior during these procedures.

It has been shown that circadian cycles influence the level of testosterone in dogs, but these fluctuations are not dependent on the light program [73]. In our study, however, due to the short duration of experiment, such changes could not be observed to a conclusive level. Elevated level of testosterone as an effect of female presence has been observed in bulls, however, only in a case of a low basal level of T, and was not observed if the basal level of T was high during stimulation [64]. Because the only group in which we found a statistically significant simple effect of samples time collection was the control group, we did not find any evidence of an increase in serum T in response to stimulation. This result was surprising, because contact of a male with female sex pheromones is expected to result in increase in testosterone level [38, 64, 74]. Cited research, however observes increased testosterone level in samples collected later (60 minutes after the exposure to pheromones in hamsters [74], and 180 minutes after contact with female in dogs [38].However in the experiment of Fukuda et al. [38], the effect of semiochemical stimulation in male dogs was observed only after the exposure to the vagina secretion of the female in estrus. When male dogs were placed with a female in estrus, however, T levels in male dogs did not increase [38]. The findings of our study are concurrent with this observation.

Considering the role of the hormones considered in our study in the context of mating behavior, the artificial and passive character of manual semen collection, without involvement of overt sexual arousal, might have elicited a much weaker stimulation of the endocrine system. It seems to be worth noticing that results obtained in humans show that masturbation provokes a much weaker impulse for secretion of hormones compared to coitus [8]. On the other hand, in stallions the OXT level increased after semen collection, which shows that the sexual arousal, connected with presence of well-expressed reproductive reflexes, that are necessary for a successful semen collection using artificial vagina, must have occurred [66].

Taking into account that in nature male dogs have usually a chance for a longer contact with the female in proestrus and estrus, following her for a couple of days (first at least 6–9 day during proestrus and then just as long in estrus), much longer stimulation, could result in much stronger observed effect. In our study male dogs had a chance only for a very short contact with a female in estrus, which was indeed enough to express behavioral arousal, however, when allowed for only a short contact with female in estrus and pheromonal stimulation, endocrinological changes could be expressed to a substantially reduced degree.

## Conclusion

The results obtained indicate that manual semen collection does not stimulate the endocrine system to a sufficient degree to elicit secretion of the evaluated hormones above the normal level. Also, a short contact with female in estrus does not markedly influence the level of the hormones. Further studies comparing manual semen collection with natural mating, allowing for the stronger and longer stimulation and expression of the full range of behavioral reaction, could better explain the role of hormones in the reproduction physiology of the males of domestic dog, including examination of the relations between semiochemicals and hormonal regulation axis in this species.

## Supporting information

S1 Dataset (XLS)
